# Estimation of the apnea-hypopnea index in a heterogeneous sleep-disordered population using optimised cardiovascular features

**DOI:** 10.1038/s41598-019-53403-y

**Published:** 2019-11-26

**Authors:** Gabriele B. Papini, Pedro Fonseca, Merel M. van Gilst, Johannes P. van Dijk, Dirk A. A. Pevernagie, Jan W. M. Bergmans, Rik Vullings, Sebastiaan Overeem

**Affiliations:** 1 Eindhoven University of Technology, Dept. of Electrical Engineering, Eindhoven, 5612 AZ The Netherlands; 2 Philips Research, High Tech Campus, Eindhoven, 5656 AE The Netherlands; 3 Sleep Medicine Centre Kempenhaeghe, Heeze, 5591 VE The Netherlands

**Keywords:** Health care, Population screening, Biomedical engineering

## Abstract

Obstructive sleep apnea (OSA) is a highly prevalent sleep disorder, which results in daytime symptoms, a reduced quality of life as well as long-term negative health consequences. OSA diagnosis and severity rating is typically based on the apnea-hypopnea index (AHI) retrieved from overnight poly(somno)graphy. However, polysomnography is costly, obtrusive and not suitable for long-term recordings. Here, we present a method for unobtrusive estimation of the AHI using ECG-based features to detect OSA-related events. Moreover, adding ECG-based sleep/wake scoring yields a fully automatic method for AHI-estimation. Importantly, our algorithm was developed and validated on a combination of clinical datasets, including datasets selectively including OSA-pathology but also a heterogeneous, “real-world” clinical sleep disordered population (262 participants in the validation set). The algorithm provides a good representation of the current gold standard AHI (0.72 correlation, estimation error of 0.56 ± 14.74 events/h), and can also be employed as a screening tool for a large range of OSA severities (ROC AUC ≥ 0.86, Cohen’s kappa ≥ 0.53 and precision ≥70%). The method compares favourably to other OSA monitoring strategies, showing the feasibility of cardiovascular-based surrogates for sleep monitoring to evolve into clinically usable tools.

## Introduction

Obstructive sleep apnea (OSA) is the most common form of sleep-disordered breathing. OSA is characterised by repetitive respiratory events during sleep, i.e. obstructions of the upper airway resulting in increased respiratory effort causing several physiological effects, such as sleep disruption and intermittent hypoxia. OSA negatively affects the quality of life and may have long-term health consequences in the untreated patient. Daytime sleepiness, fatigue, depression, metabolic dysregulation and cardiovascular complications are just a few examples of the impact on a person’s health^1^. Furthermore, the economic and healthcare relevance of sleep disordered breathing is boosted by its high prevalence^1,2^. It is estimated that 5–15% of the general population has OSA with moderate severity, meaning that respiratory events are occurring on average more than fifteen times per hour of sleep^1^. The prevalence of all sleep-related breathing disorders has increased during the last decades, driven in part by an increase in obesity^3^. Regrettably, an important part of patients with OSA remains undiagnosed, aggravating the health impact on the population. This diagnostic deficiency is caused principally by the difficulty to recognise subjects with few or subtle symptoms, by lack of awareness of the symptoms by patients and caregivers, and because suitable screening methods do not exist as of yet^4,5^.

The gold standard diagnostic procedure for OSA is a sleep-focused clinical interview accompanied by a polysomnographic recording. Polysomnography (PSG) is an overnight measurement of several physiological signals, including electroencephalography to measure sleep and a comprehensive recording of respiratory parameters. The recording is annotated by a sleep technician and evaluated, together with symptomatology, to diagnose the presence of sleep-related disorders^1,6^. The number of annotated respiratory events divided by the sleep time yields the apnea-hypopnea index (AHI). Although fraught with problems^7,8^, the canonical way of categorising OSA severity is still based on AHI thresholds of 5, 15, 30 events/h corresponding to mild, moderate and severe OSA respectively.

PSG is recorded in a clinic but polygraphy solutions for home monitoring are often used. These home-sleep-test diagnostic tools (HST) typically have a smaller amount of recorded signals, but still suffer from two main drawbacks: they are expensive (in the U.S., HST is only 10% cheaper than PSG for the provider^9^) and obtrusive^10^. These limitations preclude the possibility to use these techniques for screening purposes or for long-term monitoring of a patient. Therefore, PSG and HST may not be able to prevent the rise of undiagnosed sleep-related breathing disorders, nor can it help to improve the characterisation of the disorder due to its high night-to-night variability^11,12^. To counteract the costs and obtrusiveness of PSG and HST, several questionnaires have been developed as screening tools. Most of these questionnaires comprise questions regarding symptoms and anthropometric characteristics, such as the presence of snoring and body mass index. However, they are not responsive in all OSA patients since they are based on subjective questions and therefore cannot tackle cases with non-standard symptomatology^13,14^. In fact, questionnaires have shown an unbalanced sensitivity-specificity ratio or unsatisfactory results (e.g. the Berlin questionnaire only has 37–43% sensitivity with 84–80% specificity to screen for mild and moderate OSA respectively^15^).

In the last two decades, several tools have been proposed to enable objective OSA monitoring without incurring the costs and obtrusiveness of PSG and similar methods. These new tools combine the recording of a single physiological signal with algorithmic approaches to assess the presence of respiratory events. A large part of these unobtrusive tools employ electrocardiography (ECG) and photoplethysmography (PPG) to derive cardiovascular signals^10,16^. The features extracted from the cardiovascular signals, such as heart rate variability (HRV), can be indirectly related to the presence of sleep events. Moreover, cardiovascular information can potentially be used to assess sleep itself including sleep staging, creating a valuable and more complete picture of sleep for the clinician^17^. The interest in these methods has also increased with the rise of consumer devices monitoring cardiovascular parameters, e.g. smart-watches and fitness trackers. These consumer devices have been massively adopted by the public therefore, after proper validation, these devices could constitute a basis to mitigate the drawbacks of the standard sleep monitoring techniques^18–21^. In addition, wearable devices could be used to monitor a patient also during the day, adding a new dimension to clinical follow-up and potentially offering guidance with respect to other factors influencing OSA and sleep in general, such as weight-management programs^20^.

A sharp increase in academic publications proposing algorithms using solely cardiovascular information for OSA monitoring can be noticed from the year 2000 when the Apnea-ECG dataset of the Computing in Cardiology challenge was released online on Physionet^22–24^. The majority of these studies employ this dataset to propose new cardiovascular features or new technical approaches to monitor OSA^10^. However, the Apnea-ECG may not represent the complexity of healthy as well as disordered sleep because of the pre-selection of clean signals, the absence of sleep disorders other than sleep disordered breathing and the limited amount of recordings^10,22,25,26^. Monitoring solutions based on this dataset need to be more extensively validated and, in some case, re-developed on larger, and more complex datasets in order to unearth the potential value of unobtrusive cardiovascular monitoring as a complement to diagnostic arsenal for sleep disordered breathings.

Here, we report a novel algorithm for AHI estimation based on ECG-derived features that fully automatically provides a good representation of the gold standard AHI even in a heterogeneous sleep disordered population. The algorithm employs features that are commonly used for unobtrusive sleep analysis and are easily transferable to other wearable solutions, such as PPG^17^. The algorithm performs AHI estimation starting from the detection of 30-second epochs surrounding respiratory events (RE-epochs). The ECG-derived features are optimised with automatically selected transformations, such as different types of filters and normalisation techniques, all with the aim of increasing the detectability of RE-epochs. The best RE-epochs classifier was chosen among several models based on their performance. The AHI was calculated from the ratio between the number of detected RE-epochs and the total number of epochs analysed (i.e. epochs scored as sleep).

Importantly, we trained and validated the algorithm using a five-fold cross-validation scheme on a heterogeneous sleep-disordered population consisting of five different datasets including recordings obtained from a “real-world” clinical sleep population, not specifically selected for development of OSA monitoring algorithms. The heterogeneity of the datasets allowed us to evaluate our OSA monitoring method on several aspects: accuracy of the AHI estimation, characteristics influencing the estimation quality, the performance as an OSA screening tool as well as a severity estimation tool. Finally, we investigated the performance of AHI estimation when sleep/wake classification of epochs was done using the ECG signal as well resulting in a fully automated solution for OSA monitoring.

## Results

### Datasets and data preparation overview

We used five datasets that are considerably different from each other, generating a pooled validation set of 262 recordings (after excluding some of them due to, for instance, low signal quality as described in Recording exclusion). The St. Vincent’s University Hospital/University College of Dublin Sleep Apnea (UCD) and the Apnea-ECG datasets are available online and focus on participants with OSA^23,27^. The HealthBed dataset consists of healthy participants without sleep related complaints. The validation dataset for automated PSG scoring polysomnograms (Auto-PSG) includes participants that are representative of a clinical sleep laboratory focusing on OSA cases^28^. The Sleep and OSA Measuring with Non-Invasive Applications (SOMNIA) dataset consists of patients with a wide range of sleep disorders, among which insomnia, sleep related movement disorders, parasomnias and OSA^22^. The SOMNIA dataset is representative of the population seen in a tertiary sleep clinic, and increased the heterogeneity of the data as it includes several sleep disorders other than OSA. The main characteristics of the datasets can be found in Table 1 and more information regarding the data used in this study can be found in methods subsection Datasets. The Auto-PSG, HealthBed and SOMNIA datasets were used as training and independent validation set of each cross-validation fold, with each training and validation set balanced in terms of sleep disorders and OSA severity. The UCD and the Apnea-ECG datasets were used only as validation data in order to strengthen the performance evaluation of our methods without the risk of influencing the training with different type of annotations, i.e. the distinction between obstructive and central hypopnea in the UCD and the 60-second epochs in the Apnea-ECG dataset (more info in datasets). Our AHI estimation was based on the detection of respiratory event (RE)-epochs defined as 30-second epochs containing at least 10 seconds of a respiratory event or starting closer than 5 seconds to its ending. This definition was used to ensure that the RE-epochs closely represent the physiological response to respiratory events (more details in Respiratory event (RE)-epoch labelling). Respiratory events were labelled obstructive apnea, hypopnea (using the 4% oxygen desaturation threshold or arousal criteria^29^), central apnea and mixed apnea. As wake periods acted as confounders, AHI estimation was done by excluding the 30-second epochs annotated as wake by a human scorer or by a sleep staging algorithm.

**Table 1:** Characteristics of the pooled validation sets divided per datasets.

	Apnea-ECG	UCD	Auto-PSG	HealthBed	SOMNIA
Participants [#] (male)	68 (57)	23 (19)	50 (X)	36 (15)	**85** (44)
Age [year]	45 ± 11 [27–63]	50 ± 10 [28–68]	X	34 ± 15 [18–63]	**51** ± 16 [18–80]
BMI [kg/m2]	28 ± 6 [19–45]	**31** ± 3 [25–42]	X	24 ± 3 [18–33]	27 ± 5 [17–44]
AHI [events/h]	**29** ± 27[0–93]	16 ± 13 [4–50]	25 ± 18 [3–75]	3 ± 6[0–24]	12 ± 12[0–54]
Epochs [#]	**984** ± 62 [802−1156]	620 ± 123 [379−776]	694 ± 125 [282−893]	879 ± 70 [728−1015]	799 ± 165 [184−1050]
Obstructive apnea epochs [%]	X	6 ± 10[0–34]	**28** ± 27[0–94]	3 ± 10[0–39]	8 ± 13[0–64]
Hypopnea epochs [%]	X	83 ± 14 [53–100]	61 ± 31 [1–100]	82 ± 26 [0–100]	**85** ± 19 [1–100]
After event epochs [%]	X	10 ± 8 [1–31]	**17** ± 12[0–51]	2 ± 5[0–39]	8 ± 13[0–45]

Epoch statistics are reported after removal of (manually scored) wake epochs. “X” indicates this information could not be retrieved. Data area shown as mean ± standard deviation [range]; maximum mean values are reported in bold.

### Epoch-by-epoch classification

#### RE-epoch detection

RE-epoch detection was performed by means of a logistic regression classifier, chosen with a nested cross-validation (see Model selection and hyper-parameter tuning for detailed description). The output of a logistic regression classifier is the probability of belonging to the positive class^30^, i.e. RE-epoch in our case. The classifier had as input a set of optimised ECG-based features (HRV features + ECG-derived activity counts, described in Feature extraction) and produced the probability of each epoch being related to a respiratory event (RE-epoch probability). In order to get a epoch-by-epoch binary class prediction (RE-epoch/non-RE-epoch), the RE-epoch probability was thresholded with the aim to maximise separation of OSA severity classes (see Model selection and hyper-parameter tuning). The RE-epoch detection performance (per-subjects, average ± standard deviation) on the pooled validation sets with the human-scored wake epochs excluded was 41.31 ± 33.69% sensitivity, 91.86 ± 9.82% specificity, 52.54 ± 37.76% precision, 82.66 ± 15.66% accuracy and 0.32 ± 0.26 agreement (Cohen’s kappa^31^).

#### Influence of respiratory events and other types of events on the RE-epoch detection

**Figure 1:**
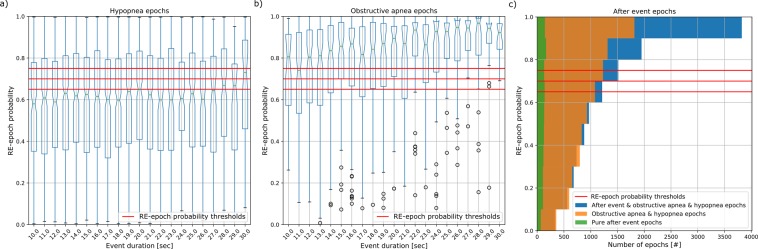
Respiratory event (RE) characteristics influencing the RE-epoch probability obtained with the epoch-by-epoch logistic regression classifier. (**a**,**b**) duration of hypopneas and obstructive apneas contained in a RE-epoch versus the RE-epoch probability of the classifier. The red lines (five lines of which two overlap to the other three) represent the RE-epoch probability threshold for the RE-epoch detection in the five cross-validation folds. (**c**) RE-epoch probability for the RE-epochs characterised by either the combined presence of an “after event” and another labelling event (shown in blue); by the exclusive presence of a hypopnea or an obstructive apnea event (orange); and by the exclusive presence of an “after event” (green). The Apnea-ECG dataset was excluded from this analysis as it does not provide event characteristics.

We investigated the influence of the type of respiratory event within an epoch on the RE-epoch probability obtained from the chosen classifier. Figure 1 illustrates the RE-epoch probability for the epoch characterised by the most common respiratory events, i.e. hypopneas and obstructive apneas. An epoch was considered to be characterised by an obstructive apnea or hypopnea depending on the longest event in the 30-second epoch; in case the epoch was closer than 5 seconds to the end of one such event the “after event” label was assigned (see Respiratory event (RE)-epoch labelling for more information). Figure 1 shows that most of the false negative RE-epochs were epochs containing a hypopnea; instead, most of the RE-epochs characterised by an obstructive event or by the “after event” were correctly detected. In addition, it can be noticed that the probability of correctly predicting a RE-epoch tends to increase with the portion of the epoch containing an obstructive apnea, while this trend was not visible for hypopneas. Epoch labelled as RE-epochs because of the “after event” can contain also contain respiratory event. Figure 1c illustrates that RE-epochs characterised by the combined presence of an “after event” and a respiratory event (labelled “after”) tend to have a higher RE-epoch probability in respect of RE-epochs characterised by the exclusive presence of a respiratory event (labelled “no after”) or of an “after event” (labelled “pure after”).

We found that also other types of sleep events influenced RE-epoch detection performance. In the datasets having detailed sleep event annotations (i.e. Auto-PSG, HealthBed and SOMNIA datasets), 16.09% of the epochs that were wrongly detected as RE-epochs contained limb movements with a duration ≥3 seconds rather than one of the positive class labelling events, therefore contributing to the false positive detection.

### AHI estimation

**Figure 2:**
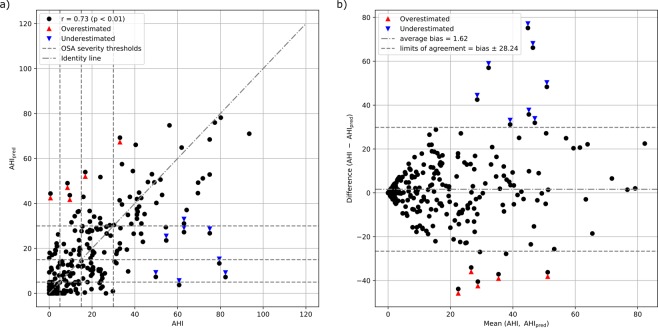
Analysis of the AHI_pred_ performance. (**a**) correlation between reference AHI versus AHI_pred_; *r* indicates the Spearman’s correlation coefficient, dashed lines delimit the canonical OSA severity classes and the dash-dotted line is the identity line. (**b**) Bland-Altman plot of the reference AHI and AHI_pred_. The bias and the limits of agreement (i.e. 1.96 times the standard deviation of the difference) are shown as events/h. Arrows indicate participants with considerably underestimated (blue) and overestimated (red) AHI (respectively AHI-AHI_pred_ >30 events/h and AHI-AHI_pred_ <−30 events/h).

The AHI was calculated for each participant from the ratio between the number of detected RE-epochs and the total number of epochs analysed (i.e. sleep epochs). Since there was not a one-to-one relationship between RE-epochs and respiratory events (e.g. one respiratory event can cover two 30-second epochs), the number of detected RE-epochs was corrected by a multiplicative coefficient obtained by linearly regressing the reference AHI with the RE-epoch ratios obtained in the training data (more details in AHI and OSA severity estimation). The AHI predicted with our method (AHI_pred_) significantly correlated with the AHI (Fig. 2a, Spearman’s rank correlation coefficient: 0.73). The average AHI estimation error for participants without OSA and those with mild, moderate and severe OSA was respectively −2.68 events/h, −2.43 events/h, 2.55 events/h and 11.22 events/h. Analysing the Bland-Altman plot, we found that the bias between AHI and AHI_pred_ was minimal, but the limits of agreement were relatively wide (Fig. 2b). There were a few participants detrimentally influencing the correlation and the limits of agreements. Eight of the 262 participants of the pooled validation set were considerably underestimated (based on the limits of agreement, difference AHI-AHI_pred_ > 30 events/h) belonging to the Auto-PSG (2), UCD (1) and Apnea-ECG (5) datasets. All these participants had an AHI ≥ 50 events/h and, on average, more than 70% of the epochs labelled as RE-epochs. Conversely, five participants were considerably overestimated (based on the limits of agreement, difference AHI-AHI_pred_ <−30 events/h), all belonging to the SOMNIA dataset. Of these five, two had a sleep movement disorders diagnosis (one comorbid with OSA, AHI = 8.53), one had an insomnia diagnosis (comorbid with severe OSA, AHI = 33.05) and two had a pure OSA diagnosis (AHI = 9.61 and 16.84 events/h). The two participants with pure OSA were characterised by a high number of hypopneas with a desaturation between 3% and 4%, i.e. not included in the events determining the RE-epochs (their AHIs with 3% desaturation threshold criteria would have been respectively 16.17 and 26.16 events/h).

Five AHI estimations for the Apnea-ECG and the UCD datasets were initially obtained because the two datasets were forced in the validation sets. These estimations were averaged to have a single AHI value per participant. We found that the performance differences between the five validation sets were negligible. For instance, the standard deviation between the validations sets was 0.019 for the Spearman’s correlation, 0.069 events/h for the bias and 0.261 events/h for the limits of agreement.

### AHI estimation for OSA screening and severity estimation

The AHI_pred_ was tested as an OSA screening tool for the three canonical OSA severity thresholds, i.e. AHI above 5, 15 and 30 events/h. The AHI_pred_ had a good agreement (Cohen’s kappa^31^ from 0.53 to 0.62) for all three OSA severities with the minimum agreement reached for the OSA cases with moderate severity. The precision (positive predictive value) and the sensitivity of our screening method decreased with an increase in severity, while the specificity followed an opposite trend (Table 2). Using the ROCs related to the three canonical OSA severities and choosing the points with the lowest Euclidean distance to a sensitivity and specificity of one, we obtained the thresholds for the AHI_pred_ at 5.32, 9.77, and 19.46 events/h, which gave the best screening performance for the three canonical OSA severities. While the optimal screening threshold for mild OSA was close to the canonical threshold, the other two severity classes had optimal thresholds approximately 30% lower than the canonical thresholds. These lower thresholds led to an increase in sensitivity, albeit at the expense of specificity and precision.

**Table 2:** Performance of the proposed ECG-based method (with manual sleep/wake scoring) for screening the three OSA severity classes, using the canonical as well as the optimal AHI thresholds.

	Minimum Severity	Cohen’s kappa	Sensitivity [%]	Specificity [%]	Accuracy [%]	Positive predictive value [%]
Using the canonical AHI thresholds	mild (AHI > 5)	0.62	84	79	82	88
	moderate (AHI > 15)	0.53	70	82	77	75
	severe (AHI > 30)	0.58	66	91	85	68
Using the optimal thresholds for AHI_pred_	mild (AHI > 5)	0.62	83	80	82	89
	moderate (AHI > 15)	0.57	83	74	78	72
	severe (AHI > 30)	0.55	83	81	82	56

**Figure 3:**
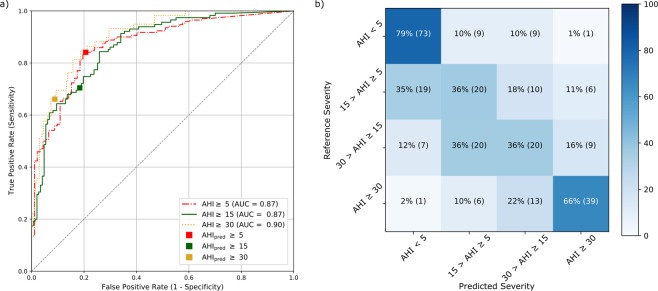
Receiver operating characteristics and confusion matrix of the AHI_pred_ for the three canonical AHI thresholds. (**a**) AUC area under each curve; square markers indicate the points in the curve where the AHI_pred_ threshold for severity classification is equal to the canonical 5, 15 and 30 events/h. (**b**) OSA severity classes obtained from the AHI (Reference Severity) and AHI_pred_ (Predicted Severity) using the canonical thresholds. In each cell, the percentage per severity is shown (also visually indicated by the colour scale) as well as the number of participants.

Using the AHI_pred_ to label each participant with an OSA severity class, our method was able in most of the cases to assign the correct class to participants without OSA (Normal label, Fig. 3b). There was a tendency to underestimate mild and moderate cases, mostly by one level of severity. The moderate and severe cases were rarely underestimated as normal.

For the screening performance, the variations between validation sets generated by the the Apnea-ECG and the UCD datasets were found to be negligible. For instance, the standard deviation between the validations sets was maximum 0.005 for the ROC AUC and maximum 0.018 for the Cohen’s kappa for the canonical AHI thresholds.

### OSA monitoring with automatic sleep/wake detection

Results so far were obtained with sleep/wake classification by a human scorer. The fully automatic version of our method was obtained by detecting and subsequently excluding the wake epochs using the ECG-based sleep staging algorithm proposed by Fonseca *et al*.^32^. This sleep staging algorithm uses a subset of the features used in our OSA monitoring solution with the addition of a feature describing the time, going linearly from 0 (start of the recording) to 1 (end of the recording). A conditional random field classifier is used to estimate the probability of each sleep stage (wake, REM, nREM1, nREM2, nREM3) and we grouped the stages to have a two class output, i.e. wake vs sleep. The algorithm was been trained and validated on different datasets. For the datasets used in this research work, the sleep/wake algorithm had an average accuracy per subject of 84%± 13% paired with a 40%± 22% sensitivity and a 88%± 13% precision for the wake periods and an agreement with the human scorer annotations (Cohen’s kappa^31^) of 0.44 ± 0.22.

Using the automatic sleep/wake scoring only led to a small decrease in performance. The AHI-AHI_pred_ correlations obtained using sleep/wake scored manually and automatically were found not statistically different (p > 0.1 for equal correlation null hypothesis using Fisher’s z transformation^33^). The fully automatic method had a lower correlation coefficient and higher limits of agreement but lower bias than the case where human scoring was used. The average AHI estimation error was similar to the one obtained with the manual scoring: respectively −2.80 events/h, −3.26 events/h, −0.30 events/h and 7.92 events/h for participants without OSA and those with mild, moderate and severe OSA. With automated annotation, seven participants showed a considerably overestimated AHI and seven a considerably underestimated AHI. Most of these participants are the same as those misdiagnosed with human sleep/wake scoring. In the considerably overestimated cases, one SOMNIA participant with insomnia comorbid with OSA (AHI = 15.97 events/h) replaced the pure moderate OSA. In addition, two more participants, belonging to the Apnea-ECG and the Auto-PSG datasets with OSA, had a considerably overestimated AHI (34.00 and 23.9 events/h respectively). For the considerable underestimation automatic sleep scoring yielded comparable results, with all the participants having severe OSA and six had an AHI ≥ 50 events/h.

Compared to human sleep/wake scoring, the fully automatic method showed an increase in sensitivity, a decrease in precision, and a mostly unchanged specificity, with a maximum decrease from 79% to 73% for screening mild OSA (Table 3). For comparison with the literature reported by Mendonça *et al*.^10^ using a non-canonical AHI threshold, the screening performance for AHI ≥ 10 was accuracy 81%, sensitivity 73% and specificity 83%.

**Table 3:** Performance of the fully automatic method for screening the three OSA severity classes.

	Minimum Severity	Cohen’s kappa	Sensitivity [%]	Specificity [%]	Accuracy [%]	Positive predictivevalue [%]
Automatic sleep/wake scoring	mild (AHI > 5)	0.57	85	73	80	84
	moderate (AHI > 15)	0.53	73	81	77	72
	severe (AHI > 30)	0.60	70	90	86	70

**Figure 4:**
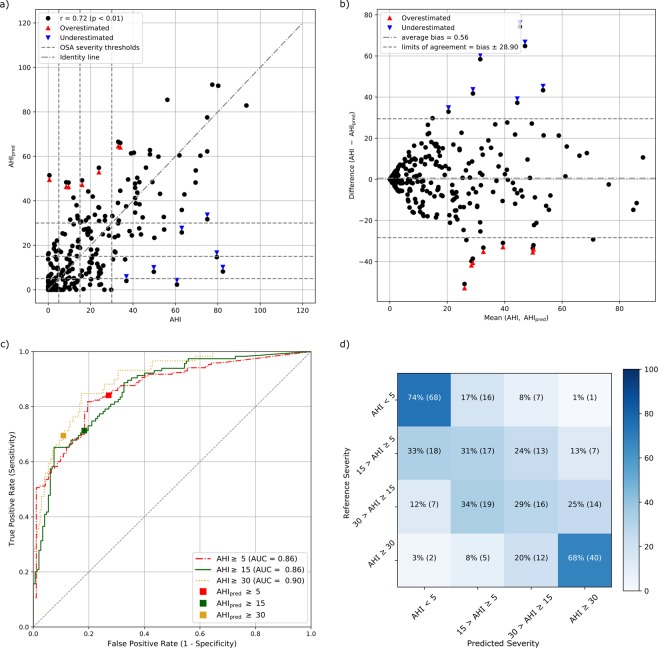
OSA monitoring performance employing the fully automated method including ECG-based sleep/wake scoring. (**a**,**b**) correlation and Bland-Altmant plot. (**c**) receiver operating characteristics describing the OSA screening for the canonical thresholds (AUC: area under the curve, squares: sensitivity-specificity points for the canonical AHI thresholds). (**d**) confusion matrix of the OSA severity (percentage per reference label and number of participant in parenthesis).

The OSA severity estimation performance obtained with the automatic method compared well with the manual sleep/wake scoring, with a moderate increase in the overestimation of OSA severity, especially for the participants without OSA (AHI < 5, Fig. 4d).

## Discussion

Here, we present an AHI estimation method based on optimised ECG-derived features with cross-validation in a heterogeneous sleep-disordered population.

We showed that the AHI predicted with this method is a good representation of the gold standard AHI, even in the complex ensemble of the included datasets. Moreover, our algorithm not only provided an adequate estimation of the AHI when epochs were manually scored as sleep or wake, but even when using an automatic ECG-based surrogate sleep/wake classification method. Only 3% of the epochs scored as wake, both in case of the human and automatic scoring, were related to a respiratory event, therefore the AHI underestimation caused by their exclusion can be considered negligible.

In a small number of participants the AHI was considerably over- or underestimated. Considerable AHI overestimation mainly occurred in participants with comorbid sleep disorders that have an impact on cardiovascular features, such as insomnia and sleep-related movement disorders^34–36^. The effect of these sleep disorders on the features is also confirmed by the relatively large amount of epochs with movements that are misdetected by our method as RE-epochs. This misclassification can be explained by the similarity of the autonomic nervous system response between respiratory events and limb movements and their occasional co-occurrence^37^. Especially the latter weakens the link between cardiovascular features and respiratory events, even in the presence of movement related features, such as ECG-derived activity counts in our case. In the fully automatic method, AHI overestimation occurred a little more often (seven participants instead of five), probably due to misdetection of wake periods, leading to an increased amount of non-RE-epochs with a higher autonomic nervous system activity. The reference AHI of the overestimated participant was heavily dependent on the oxygen desaturation threshold chosen to score the hypopneas: the AHI obtained with a 3% desaturation threshold was almost double than the AHI obtained with the 4% desaturation threshold. This is in line with the literature and rekindles the discussion regarding what would be the best definition for this type of respiratory events, also from a cardiovascular response perspective^29^. The overestimation of AHI in certain cases does not necessarily diminish the potential of our method: a high AHI_pred_ could trigger a clinician to the presence of abnormal sleep and lead to a more thorough sleep investigation.

The cases in which our method considerably underestimated the AHI may be explained by the datasets used to train our method, i.e. the SOMNIA, the HealthBed and the Auto-PSG datasets. These datasets have a lower number of extremely severe OSA cases compared to for example the Apnea-ECG dataset. This might have biased the epoch-by-epoch classifier, the RE-epoch probability thresholds choice and the feature optimisation towards lower AHI participants. Especially feature optimisation could have been affected by this: the per-subject normalisation selection can disregard the flattening of the features generated by some normalisation (e.g. Z-score) occurring when most of the recording is characterised by respiratory events (such as those with an AHI > 50 events/h). We hypothesise that the cases with an underestimated AHI reflect a limitation of the datasets avaible rather than of the method per se. If so, this may be ameliorated by including more extreme OSA cases in the algorithm training.

The significant correlation between the AHI and its estimation, for both versions of our method, is supported by the average AHI estimation error for the different OSA severity thresholds. Leaving the severe OSA aside for the reason previously mentioned, the average estimation error for participant without OSA and those with mild and moderate OSA was small and the sign was mostly in agreement, with only the moderate OSA having a negligible change to AHI underestimation from the minor overestimation of participants without and with mild OSA.

When our AHI_pred_ was employed as screening tool, its performance was superior to three of the most used questionnaires, independently from the sleep/wake scoring technique applied. As an illustrative comparison, the STOP-Bang questionnaire, the Berlin questionnaire and the Multivariable Apnea Prediction score showed lower sensitivity and specificity for screening mild and moderate cases than our method when validated in large general population even less complex as ours^15,38,39^. Our method showed a tendency to under-screen severe OSA cases, but most of these were labelled as moderate OSA and therefore still tagged as participants that require further clinical investigation.

The screening performance of our method can be compared against other ECG-based screening methods as well. In a recent review on OSA monitoring approaches, two studies proposing ECG-based OSA screening methods were found to be tested on datasets with more than 100 participants^10,40,41^. Using an AHI screening threshold of 10 events/h, Roche *et al*. reported an accuracy of 91% with a 92% sensitivity and 90% specificity on 147 participants while Gutierrez *et al*. showed 72% accuracy with 80% sensitivity and 59% specificity on 188 participants. The performance of our method lies between these two studies. However, while our method is validated on a heterogeneous pool of datasets consisting of 262 participants, the population of these other studies consisted of participants specifically referred to the hospital for a suspicion of OSA. This could have biased results towards higher agreement, as Roche *et al*. suggested as well^40^.

The screening performance of our method varied for different severity classes: a lower sensitivity and positive predictive value were achieved when screening for moderate and severe OSA in comparison with mild OSA. The reason for this performance variation can be found in the choice of the RE-epoch probability thresholds for the RE-epoch detection combined with our dataset. Since the thresholds were chosen to maximise the OSA severity class separation, our method priorities a reduction of false positives at the cost of a decreased amount of true positives in order to correctly assign the OSA severity class to the most prevalent OSA cases, i.e. AHI < 5 events/h with and without other sleep disorders (Table 1). In addition, the lowest sensitivity and positive predictive value in screening the severe OSA cases are due to participant with a considerably underestimated AHI having a larger weight in the performance calculation.

The under-screening of moderate and severe OSA cases can be corrected by lowering the screening thresholds applied to the AHI_pred_. In fact, by choosing optimal thresholds for AHI_pred_, the moderate and severe OSA screening results are aligned with the mild cases (Table 2).

The screening improvement obtained with the optimised thresholds can be related to the presence of the Apnea-ECG dataset only in the validation set: with its underestimated severe OSA cases and moderate OSA cases without comorbidities, the Apnea-ECG dataset influenced the optimal AHI screening threshold to lower values. In fact, the optimal screening thresholds reduced the specificity of our system making it less resilient to participants with overestimated AHI, i.e. principally participants with sleep disorders.

Even though the differences in screening performance between the fully automatic and the manual sleep/wake scoring versions of our method were minimal, using an automatic sleep/wake scoring had the effect of decreasing specificity, while increasing sensitivity. This effect was clearer for mild OSA cases due to the higher impact of sleep/wake misclassifications, especially for participants with other sleep disorders. However, the false positive screening for mild OSA of participants with sleep disorders other than OSA is not alarming in a clinical scenario. On the contrary, it can help to prompt further investigation in patients with other clinically relevant sleep conditions. Instead, the misclassification of wake as sleep had the advantage of increasing the screening sensitivity of our algorithm. The inclusion of wrongly labelled sleep epochs leads to an increased amount of epochs detected as RE-epochs and, therefore, to an increase of AHI_pred_. Using the automatic sleep/wake scoring algorithm practically creates a compromise between the confounding effect of wake epochs (increase false positive RE-epochs) and their representative value in case of disordered sleep. Even though this detection might not be related to respiratory events, this can be interpreted as a consequence of disturbed sleep and therefore easing the screening task of our method. The screening results obtained could probably be further improved by combining our method with questionnaires, similarly to the combination of the Multivariable Apnea Prediction score with HST presented by Gurubhagavatula *et al*.^39^.

The OSA severity estimation obtained by the AHI_pred_ was in line with the reference, especially for participants without OSA. Importantly, only 8% of the participants with moderate or severe OSA were classified as normal, ensuring that the participants with the two highest severities are mostly recognised as having at least a certain degree of OSA.

HST currently are a widely accepted alternative for the PSG measurement and it is interesting to note that the AHI estimation performance of our method is comparable with typical HST automatic AHI estimation. As an example, in comparison with the Embletta and the ApneaLink HST tested by Aurora *et al*.^42^, our method had larger limits of agreement, but a lower bias. When the AHI_pred_ was used for OSA severity estimation, our method gave a slightly better performance than the Embletta HST. The percentage of underestimated cases is lower in our research for all the three OSA severities, but we had a higher overestimation, probably influenced by the inclusion of sleep-disordered participants in our data.

The RE-epoch detection part of our method showed good outcomes for the population analysed (high accuracy with a fair agreement with the reference) even though it was not the main focus of our tool. In fact, specificity was favoured over sensitivity to account for the elevated presence of confounding factors due to disordered sleep and events associated with it. Furthermore, also the precision metric was affected by the presence of confounding factors and their detrimental effect was magnified by the high imbalance between the classes (RE-epoch vs non-RE-epoch). For comparison with the literature on the Apnea-ECG dataset^10^, the performance obtained on this dataset (calculated on the entire amount of classified epochs) was 82.70 sensitivity, 80.30 specificity, 73.02 precision, 81.24 accuracy and 0.61 agreement. The results of RE-epoch detection in the Apnea-ECG dataset were in line with these literature^10,22^, which is remarkable considering that our method was trained on a different population, using the entire Apnea-ECG dataset as a hold-out set, and that our focus was AHI estimation rather than RE-epochs detection. The variance of the results due to forcing the Apnea-ECG and UCD datasets in the validation sets was negligible, indicating that the method was minimally influenced by changes of the training data.

Having several datasets with extensive sleep annotations allowed us to investigate at the event level which factors played a key role in AHI estimation. We noticed that most of the RE-epochs characterised by obstructive apneas were correctly detected. In addition, their RE-epoch probability seems to be proportional to the duration of the event in the epoch (Fig. 1b). The relationship between event duration and RE-epoch probability can be explained with a proportionality between cardiovascular response duration and event duration: longer events influence a larger amount of the ECG signal and, therefore, stronger affect the features calculated on windows of this signal. In the case of the hypopnea-characterised epochs, we hypothesise that this relationship could be hidden by the milder cardiovascular response of the hypopneas as compared to the obstructive apneas.

Hypopnea-characterised RE-epochs were the most difficult to detect, with more than 50% of them having a probability lower than the detection thresholds (Fig. 1a). The more complex detection of hypopneas with respect to obstructive apneas is in line with the literature. For instance, Punjabi *et al*.^28^ showed that, even when the AHI is estimated with a HST, the reference and estimated hypopnea indexes were less correlated when compared to the obstructive apnea indices. The hypopnea-epoch misclassification can be explained with the higher difficulty in discerning, from a cardiovascular perspective, hypopneas from other types of confounding events, such as respiratory-related arousals and limb movements^35,43^. In fact, rather than having a low RE-epoch probability for hypopnea-characterised RE-epochs (median ≥ 0.6 with 25th percentile ≈0.4), our method had an elevated RE-epoch probability thresholds (minimum 0.65). This indicates that, in order to better estimate the severity class of the participants in the training set, our method had to sacrifice the hypopnea detections in favour of increased resilience to other factors. However, it was able to compensate for these false negatives and deliver an AHI_pred_ in line with the reference AHI, especially considering the predominant presence of hypopnea-characterised epochs in our pool of datasets (Table 1).

The RE-epochs characterised by the “after event” in combination with an obstructive apnea or hypopnea were generally correctly classified by our method (Fig. 1c) probably because the end of a respiratory event is usually characterised several events (e.g. the resumption of normal breathing, arousal and a bradycardia-tachycardia behaviour) that are easier to recognise respect to, for instance, the first 10 seconds of a respiratory event^1,16^.

The advantages of having a heterogeneous sleep-disordered population are accompanied with some disadvantages. The sleep disorder heterogeneity of the data limited us to cross-validate our method rather than validating it on a separate hold-out set. Future validations of the algorithm will also aim to characterise in detail the behaviour of our algorithm, for instance by evaluating the performance for different sleep stages obtained from manual annotation and sleep staging algorithms. The epoch misclassification concomitants with the presence of limb movements indicates that our approach could be less suited for patients with sleep movement disorders. Therefore, future research should focus on reducing this misclassification, for instance by using activity counts obtained from an accelerometer rather than an ECG movement surrogate. Even though it is currently the clinical standard metric to monitor OSA, the AHI is just one representation of the disorder. Consequently it is intrinsically limited to describe a complex, and yet to be fully understood, disorder such as OSA^7,8^. However, the AHI is valuable information that contributes to the overall clinical picture of a patient. Therefore, we decided to focus on the current clinical standard, leaving to future studies the possible investigation of using our method to derive metrics to monitor OSA other than AHI. The exclusion criteria related to technical and physiological aspects of the ECG signal (described in Recording exclusion) represent another limitation. Low coverage and sensor malfunctioning can be overcome by the possibility to measure multiple nights with an unobtrusive implementation of our method, but our method cannot handle participants with a high presence of irregular beats because it is largely based on HRV features. However, the cardiovascular signal, such as ECG and PPG, can inform the clinician on the presence of such irregular beats and, therefore, direct those patients to more standard sleep analysis.

We selected our epoch-by-epoch classifier among classifiers that are simpler than the latest developments in the machine learning field for sleep^44,45^. While this allowed to minimise hyper-parameter tuning and computational power required for training, nevertheless it limits the information that can be inferred from our features. More complex classifiers combining both feature optimisation and classification will be part of future research. Future research will also aim to translate this method for other type of sensors, like it has been done for sleep staging with wrist-worn PPG^17^. This is likely to be possible because the features chosen are device agnostic, i.e. they can be derived from all the devices able to extract inter-beat intervals and movement information. The implementation of our method on devices like smartwatches or ECG patches will enable a fully automatic, unobtrusive and long-term OSA monitoring.

## Conclusion

Here, we propose a method providing an AHI estimation in a heterogeneous sleep-disordered population using a cardiovascular signal that is collectable in an unobtrusive manner. The method shows good correspondence with AHI values obtained with conventional, obtrusive methods. We validated our method in a challenging scenario, using data from multiple sleep disorders, with a high prevalence of hypopnea events, and recordings annotated in different sleep centres over a wide period of time. Results showed that the estimated AHI can be used both for OSA screening and for OSA severity estimation. Thanks to the increased availability of sleep-related datasets and the rapid evolution of machine learning techniques, we believe that in the future OSA monitoring by means of unobtrusive cardiovascular signals has the potential to evolve from a challenging research topic to a clinically accepted tool.

## Methods

### Datasets

This research work employed five datasets collected by different teams and sleep centres, using various set-ups.

The UCD dataset^27^ contains 25 overnight PSG recordings of adults with suspected sleep-related breathing disorders collected at the Sleep Disorders Clinic of the St Vincent’s University Hospital (Dublin, Ireland). For our analysis, we used the signals collected from the ECG modified lead V2 (sampling frequency 128 Hz) and the respiratory event annotations (obstructive apnea and hypopnea, central apnea and hypopnea, and mixed apnea). The obstructive hypopnea and central hypopnea were grouped together in a single hypopnea class since the differentiation of these two events still lacks clinical consensus^46^.

The Apnea-ECG dataset^23^ contains 70 overnight recordings that were assembled together for the Computing in Cardiology challenge of 2000. The goal of the challenge was to detect 60-second epochs containing respiratory events and to categorise the recordings as normal or apneic (based on an AHI threshold of 10 events/h). For our analysis, we used the 60-second annotation (i.e. apneic/normal epoch) and the ECG signal (sampling frequency 200 Hz) provided. The events determining the 60-second annotations were scored according to the AASM 1999 guidelines. As our algorithm predicts RE-epochs with a 30-second epoch resolution. We split each epoch of the Apnea-ECG dataset into two epochs with the same label as the original one. We used the Apnea-ECG dataset as a whole without the train and test set separation adopted in the Computing in Cardiology challenge.

The Auto-PSG dataset^28^ consists of 97 overnight PSG recordings of adults acquired in four sleep centres in the United States. The data collection protocol was approved by the institutional review board of each clinical site, as described in the paper of Punjabi *et al*.^28^. The dataset can be sub-dived in three types of PSG recordings: 31 diagnostic PSG, 35 CPAP titration nights, and 31 split-nights (half night diagnostic, half night titration). This dataset was annotated manually by four different certified scorers and by the automated Philips Somnolyzer system^28^ according to the 2007 AASM criteria. We used the modified lead II ECG signal (sampling frequency 200 Hz) and, as reference, the annotation done by the Somnolyzer system after this was reviewed by one of the human scorers. Detailed information regarding this dataset can be found in the paper published by Punjabi *et al*.^28^.

The HealthBed dataset consists of 40 recordings collected at the Sleep Medicine Centre Kempenhaeghe (Heeze, The Netherlands). The participants were healthy adults without sleep complaints or disorders or other medical or psychiatric comorbidity. Each recording consists of a full PSG with clinical annotations (hypnogram and sleep events AASM 2012 guidelines^29^ with 3% desaturation criteria for hypopneas) scored by an expert sleep technician. We used the modified lead II ECG signal (sampling frequency 512 Hz) as well as the annotations. The HealthBed study was reviewed by the medical ethical committee of the Maxima Medical Center (Eindhoven, the Netherlands. File no: W17.128), all participants gave written informed consent.

The SOMNIA dataset used is part of an ongoing data collection done in the Sleep Medicine Centre Kempenhaeghe (Heeze, The Netherlands), including unselected patients scheduled for a routine diagnostic PSG. A total of 103 recordings were selected targeting a study population of at least 50% of patients having an OSA diagnosis (with an approximately uniform severity distribution). Exclusion criteria were predominant central sleep apnea and Cheyne-Stokes breathing conditions and recordings while using CPAP or CPAP titration. The dataset comprised of the following participants: 48 patients with OSA (of which 32 without any comorbidity, 10 with insomnia disorders and 4 with sleep movement disorders and 2 with other comorbid sleep disorders), 27 with an insomnia diagnosis (of which 13 without sleep comorbidity), 10 with a sleep movement disorder diagnosis (of which 4 without sleep comorbidity), 7 with parasomnia diagnosis (of which 4 without any comorbidity), 8 with other sleep disorders (of which two with circadian sleep disorder, three with behaviourally induces insufficient sleep syndrome and three with chronic fatigue syndrome) and one without any sleep disorder. For this study, only the ECG modified lead II (sampling frequency 512 Hz), the annotations done by expert sleep technicians from the Sleep Medicine Centre Kempenhaeghe, and the patient diagnosis were used. Also for this dataset, the AASM 2012 annotation guidelines were followed. The SOMNIA study was reviewed by the medical ethical committee of the Maxima Medical Center (Eindhoven, the Netherlands. File no: N16.074), and all participants gave written informed consent.

All the studies met the ethical principles of the Declaration of Helsinki, the guidelines of Good Clinical Practice and the current legal requirements. The protocol for data analysis was approved by the Medical Ethical Committee of the Kempenhaeghe hospital (number 06.17) and by the Philips Institutional Review Board (Internal Committee on Biomedical Experiments, identification numbers ICBE-2-14791 and ICBE-2-18859).

The UCD, the HealthBed and the SOMNIA datasets applied the 3% oxygen desaturation rule for the scoring of hypopneas in absence of arousals. In our research, the hypopneas that were not followed by an arousal and had a desaturation smaller than 4% were ignored. This was done to harmonise the hypopnea scoring rule between the datasets and to favour the hypopnea desaturation threshold with a higher impact on the cardiovascular system^47^.

### Recording exclusion

We removed some recordings from the datasets based on three criteria: technical faults, too low coverage and presence of irregular heartbeats.

A total of 15 recordings were excluded for technical faults, such as missing signals or annotations. Of these, seven belonged to the Auto-PSG dataset, one to the HealthBed and seven to the SOMNIA dataset.

Four recordings were excluded because 70% of the features could not be calculated for more than 30% of the duration of the recordings. The reasons could have been a low quality of the ECG signal or errors detecting heartbeats. Of these, two belonged to the HealthBed dataset and two to SOMNIA dataset.

For this study, we considered an inter-beat interval (IBI) and its precedent IBI related to an irregular beat, i.e. ectopic, when the relative duration of the first with respect to the second was lower than a certain threshold (see subsection Features extraction for more details). These IBIs were excluded, and in some case interpolated, to perform the HRV analysis^48^. However for short term HRV estimation, the presence of 5% of removed IBIs over the total amount of IBIs, whether related to artefacts or irregular heart-beats, can already cause a significant deviation from the actual HRV values^49^. Since our method is strongly dependent on heart rate dynamics, we decided to exclude recordings with a number of IBIs related to ectopic beats higher than 3% of the total number of IBIs and to leave the task of extending our findings on participants with a higher amount of irregular beats for future research. As consequence, a total of 26 recordings were excluded due to a substantial presence of irregular heartbeats. Of these recordings, two belonged to the Apnea-ECG dataset, two to UCD, one to HealthBed, twelve to Auto-PSG and nine to SOMNIA.

### Cross-validation

We performed a nested cross-validation for algorithm development and evaluation. The outer cross-validation (outer CV) was used to obtain the AHI estimation results while the inner cross-validation (inner CV) to select the epoch-by-epoch classifier and parameters. Both the inner and the outer CV are done at the participant level in order to have each participant exclusively in the training or in the validation of each fold. The outer CV was a conditioned 5-fold cross-validation: depending on recording characteristics, some participants were forced in the training set and some others in the validation set of each fold. The participants of the Auto-PSG dataset undergoing titration were forced in the training set of each fold since they do not represent the population on which an unobtrusive OSA monitoring method would be applied. However, the titration subset of the Auto-PSG dataset increases the variability of the data and therefore can help, for instance, improving the generalisation of trained algorithms.

The UCD and the Apnea-ECG datasets were forced in the validation set of each outer CV fold due to the different type of annotations, i.e. the distinction between obstructive and central hypopneas in the UCD and the 60-second epochs in the Apnea-ECG. This yielded the advantage of having the full Apnea-ECG dataset as a hold-out set, so results can be compared with the literature. Since each outer CV fold yield a different performance for these two online datasets, the results presented in this study are averaged across the outer CV folds.

The recordings belonging to the SOMNIA, HealthBed and Auto-PSG datasets were divided into training and validation sets for each outer CV fold, keeping the same distribution of the prevalence of the main sleep disorders, i.e. patients with AHI below/above 15 and the presence of insomnia or sleep movement disorder. The HealthBed and the Auto-PSG (without titration subjects) datasets did not include subjects with insomnia or sleep movement disorders, therefore only the AHI value was taken into consideration for balancing training and validation sets for each fold.

The outer CV folds had an average of 161 recordings for training and of 129 for validation (varying in each fold depending on the balancing of the two sets). This results in a total amount of 262 unique validation recordings pooled from all the outer CV folds.

### Respiratory event (RE)-epoch labelling

Most of the published studies regarding epoch based apnea monitoring using cardiovascular features employ a 60-second based segmentation of the recordings, probably due to the influence of the broadly used Apnea-ECG database^22,23^. However, a respiratory event can have a minimum duration of 10 second, which is far shorter. In addition, the 60-second epoch labelling is in dissonant with the 30-second epoch approach used for clinical sleep staging and annotation. 30-second epochs were considered related to a respiratory event if they contained at least 10 seconds of a respiratory event or if the previous epoch had a respiratory event closer than 5 seconds to its end (“after event” characterised RE-epochs). The first criterion was chosen in order to guarantee enough influence of the respiratory event on the extracted features in the labelled epoch. The second criterion was chosen to compensate for the elongated influence of a respiratory event on the cardiovascular system. Most of the respiratory events are followed by arousals and/or oxygen desaturations that are prone to drive cardiovascular behaviour after the respiratory event. According to the literature, most arousals and desaturations after respiratory events have a duration of, respectively, 10 seconds and 30 seconds^34,47,50–52^. A 5 seconds threshold allows labelling the RE-epochs by ensuring that they contain the apex of the cardiovascular response (e.g. lowest desaturation or peak in heart rate), without including in the RE-epochs those characterised by the slow returning to normal sleep.

### Feature extraction

The prediction of RE-epochs was based on features extracted from the ECG signal which are commonly used in literature in the domain of sleep monitoring. A total of 123 features were extracted from the ECG signal of each recording; Table 4 gives an overview of the features together with a reference for their description. Each feature was calculated over a certain time window, dependent on the feature type, and assigned to the central 30-second epoch indicating the epoch class, i.e. RE- or non-RE-. All features, except the ECG-derived actigraphy, require the calculation of the IBIs from the ECG signal. The IBIs were computed as the time distance between each pair of consecutive R-peaks in the ECG signal. The R-peaks were detected by using an Hamilton-Tompkins detector in combination with an algorithm to refine the peak localisation^53,54^. IBIs with a value longer than 2 seconds or shorter than 0.5 seconds were rejected because they were considered not physiologically normal for a sleep recording, even when respiratory events are present^55,56^. IBIs were also rejected in case they were related to ectopic beats. An IBI and its preceding were excluded if the ratio between the two was larger than 1.5. For example, this can be seen as a drop in heart rate from 60 bpm to 40 bpm in just 1.5 second. This criterion takes advantage of the combination of having a shorter IBI before the ectopic beat and a longer IBI after the ectopic beat. In our research, two conditions had to be met to calculate a feature for a certain epoch: the IBIs have to cover at least half of the time window (feature specific) and at least 1/6 of the central 30 seconds of the window, i.e. the epoch to which the feature is assigned.

**Table 4:** Overview of the extracted features.

Features type	Number	Described in	Window size [sec]
Arousal probability	5	^61^	120
HRV frequency analysis	18	^48,62,63^	300
Adapted HRV frequency analysis	6	^64^	300
Detrended fluctuation analysis	5	^56,65,66^	360
Progressive detrended fluctuation analysis	1	^67^	60
Windowed detrended fluctuation analysis	1	^68^	360
High-frequency pole analysis	2	^69^	300
Multi-scale entropy	20	^70–72^	540
Local phase coordination	7	^73–75^	150 and 90
HRV time analysis and statistics	37	^48^	300
Sample entropy	1	^76^	300
Visibility graph analysis	13	^77–80^	210
Hilbert transformation analysis	6	^81^	300
ECG derived activity counts	1	^82^	30

Each feature was calculated using the methods proposed in the respective original methodological paper(s).

### Feature optimisation framework

For each training set of the outer CV folds, the 123 features extracted from the ECG signal were optimised with respect to their discriminative power between RE- and non-RE-epochs. The optimisation framework consisted of a greedy search of the best sets of feature transformations, applied per recording, following a graph defining the connection rules between each transformation. An exhaustive list of all the transformations and their connection can be found in Table 5. The graph was created to avoid unnecessary computation or possible errors. For instance, it is not logical to clip the outliers before changing the overall feature distribution, therefore, the graph does not connect these two transformations in this specific order. The selection criteria to determine the best set of transformations for each feature was based on absolute mean distance (AMSD):

1 $$AMSD=|\frac{{\mu }_{RE-epochs}-{\mu }_{non-RE-epochs}}{{\sigma }_{RE-\text{}+\text{}non-RE-\text{}epochs}}|.$$

here *μ*_*RE*−*epochs*_ and *μ*_*non*−*RE*−*epochs*_ indicate the average of the transformed feature values, across all the recordings, corresponding to RE- and non-RE-epochs and *σ*_*RE*− + *non*−*RE*− *epochs*_ indicates the standard deviation of the feature. Starting from the original feature, the optimisation algorithm applied all the transformations and selected the best transformation. Then, the algorithm iteratively applied another set of transformations, following the rules defined by the graph, and selected the best one of each iteration. The optimisation algorithm stopped iterating when additional transformations did not improve the AMSD or when a series of five transformations was reached. Different feature selection techniques have been evaluated, for instance, techniques based on the correlation between features and RE-epochs or on the cross-validated calculation of the AMSD. However, none of the evaluated methods was able to outperform the direct usage of optimised features.

**Table 5:** List of transformations applied to optimise the ECG-derived features.

Transformation	Group	Description	Parameters	Input groups
None	0	no transformation	—	raw feature
Winsor	1	percentile based clipping	[1–99] and [5–95] percentiles	0, 4, 6, 7, 8, 9
Median	2	removal of the median value	—	0, 1
Z-score	3	removal of the mean value and standard deviation scaling	—	0, 1, 4
Percentile	3	normalisation of the percentiles to the [0, 1] range	—	0, 1, 4
Amplitude	3	amplitude scaling	—	0, 1, 4
Box-Cox	4	Box-Cox transformation with + post Z-score normalisation	—	0, 1, 6, 7, 8, 9
Histogram	4	equalisation of the feature based on a 10-bins histogram	—	0, 1, 6, 7, 8, 9
Quantile	4	quantile normalisation with a reference distributions	normal, exponential and uniform	0, 1, 6, 7, 8, 9
Tukey-Ladder	4	Tukey-Ladder transformation	—	0, 1, 6, 7, 8, 9
Time-shift	5	features shifting in time	−1 and +1 epoch	0
MAD	6	windowed mean absolute deviation	windows of 7, 15, 23 and 31 epochs*	0, 5
SD	6	windowed standard deviation	windows of 7, 15, 23 and 31 epochs*	0, 5
Abs	6	absolute value calculation	—	0, 5
Diff	6	derivative calculation	first derivative	0, 5
Int	6	cumulative summation after mean removal	—	0, 5
Average	7	running mean filtering	windows of 7, 15, 23 and 31 epochs*	0, 5, 6
Median	7	running median filtering	windows of 7, 15, 23 and 31 epochs*	0, 5, 6
LPF	7	filtering with a second order low pass Butterworth filter	0.25, 0.5 and 0.75 normalised frequency	0, 5, 6
Subtract Median	8	windowed median subtraction	windows of 7, 15, 23 and 31 epochs*	0, 5, 6, 7
HPF	8	filtering with a second order high pass Butterworth filter	0.25, 0.5 and 0.75 normalised frequency	0, 5, 6, 7
Exponential	9	exponentiation of the feature	square and square root	0, 5, 6, 7, 8
Log	9	logarithmisation of the feature	natural logarithm	0, 5, 6, 7, 8

Each transformation belongs to a group and the connection between groups, i.e. the graph, are described by the “Input groups” column. *the windows were applied with respect to each epoch in a backward, forward and centred manner.

### Model selection and hyper-parameter tuning

Five inner CVs, one for each outer CV fold, were used to select a final model and its hyper-parameters. Each inner CV consisted of a 4-fold cross-validation on the training data of the corresponding outer CV fold. The models that were investigated consisted of linear classifiers based on log loss (i.e. logistic regression), Huber loss, and linear and quadratic discriminant analysis. In each outer CV fold, the models’ performances were graded based on the average area under the curve of the precision-recall plot obtained with the inner CV. The model with the highest average inner CV performance among each outer CV folds was selected as the final model. The final selected model was a logistic regression classifier with both an l1 and l2 regularisation. This model was trained with a stochastic gradient descent approach on the training set of each outer CV fold. The number of RE- and non-RE-epochs was unbalanced and, specifically, was leaning towards the latter. Therefore, before training the models, the training data were over-sampled for the minority class (RE-epochs) using a synthetic minority over-sampling technique^57^ and cleaned using an edited nearest neighbours method in order to remove noisy synthetic samples introduced by the oversampling of outlier populated areas of the feature space^58^.

The trained model predicted the probability of each epoch to be a RE-epoch. This probability, ranging from zero to one, was thresholded in order to assign a class to each epoch. The choice of the probability thresholds was done by maximising the Cohen’s kappa coefficient calculated between the estimated and the reference OSA severity class (after the AHI was estimated) on the training data of each outer CV fold. The following equations were applied both to compute the RE-epoch probability threshold and, once this was obtained, to predict the AHI:

2 $$j={{\rm{j}}}^{{\rm{th}}}\,{\rm{outer}}\,{\rm{CV}}\,{\rm{fold}},$$

3 $${\beta }^{train}(j)={\rm{T}}{\rm{h}}{\rm{e}}{\rm{i}}{\rm{l}}-{\rm{S}}{\rm{e}}{\rm{n}}\,{\rm{R}}{\rm{e}}{\rm{g}}{\rm{r}}{\rm{e}}{\rm{s}}{\rm{s}}{\rm{i}}{\rm{o}}{\rm{n}}({\rm{A}}{\rm{H}}{\rm{I}}(j),\text{}\frac{{\sum }_{i=1}^{{N}_{{\rm{e}}{\rm{p}}{\rm{o}}{\rm{c}}{\rm{h}}}(j)}{\rm{r}}{\rm{e}}{\rm{a}}{\rm{l}}\,{{\rm{e}}{\rm{p}}{\rm{o}}{\rm{c}}{\rm{h}}}_{i}\,{\rm{c}}{\rm{l}}{\rm{a}}{\rm{s}}{\rm{s}}(j)}{{N}_{{\rm{e}}{\rm{p}}{\rm{o}}{\rm{c}}{\rm{h}}}(j)}),$$

4 $${{\rm{A}}{\rm{H}}{\rm{I}}}_{{\rm{d}}{\rm{e}}{\rm{r}}}^{{\rm{t}}{\rm{r}}{\rm{a}}{\rm{i}}{\rm{n}}}(j)={\beta }^{train}(j)\times \frac{{\sum }_{i=1}^{{N}_{{\rm{e}}{\rm{p}}{\rm{o}}{\rm{c}}{\rm{h}}}}{\rm{r}}{\rm{e}}{\rm{a}}{\rm{l}}\,{{\rm{e}}{\rm{p}}{\rm{o}}{\rm{c}}{\rm{h}}}_{i}\,{\rm{c}}{\rm{l}}{\rm{a}}{\rm{s}}{\rm{s}}({\rm{j}})}{{N}_{{\rm{e}}{\rm{p}}{\rm{o}}{\rm{c}}{\rm{h}}}(j)}.$$

5 $${{\rm{A}}{\rm{H}}{\rm{I}}}_{{\rm{p}}{\rm{r}}{\rm{e}}{\rm{d}}}^{{\rm{t}}{\rm{r}}{\rm{a}}{\rm{i}}{\rm{n}}}(j)={\beta }^{train}(j)\times \frac{{\sum }_{i=1}^{{N}_{{\rm{e}}{\rm{p}}{\rm{o}}{\rm{c}}{\rm{h}}}}{\rm{p}}{\rm{r}}{\rm{e}}{\rm{d}}{\rm{i}}{\rm{c}}{\rm{t}}{\rm{e}}{\rm{d}}\,{{\rm{e}}{\rm{p}}{\rm{o}}{\rm{c}}{\rm{h}}}_{i}\,{\rm{c}}{\rm{l}}{\rm{a}}{\rm{s}}{\rm{s}}({\rm{j}})}{{N}_{{\rm{e}}{\rm{p}}{\rm{o}}{\rm{c}}{\rm{h}}}(j)}.$$

The AHI_der_^train^ is the AHI derived by using the ground-truth epoch labelling, while AHI_pred_^train^ is the AHI obtained by using epoch class predicted by the trained models. The Theil-Sen regression^59^ was chosen because it is more resilient to outliers due to, for instance, participants with long respiratory events and consequently a larger number of RE-epochs than the number of respiratory events. All these steps were accomplished on the training set of each outer CV fold, blindly from the validation set, in order to guarantee unbiased results.

While the training of each model was done by including all the epochs, the choice of the RE-epoch probability thresholds and the estimation of β^*train*^ were done after the removal of the wake epochs.

### AHI and OSA severity estimation

The transformed features, the models with their hyper-parameters and RE-epoch probability thresholds were used to classify the 30-second epochs of the validation set of each outer CV fold. The classification output was then filtered to remove wake epoch and remaining epochs were used to calculate the AHI_pred_ for each participant with the same strategy as applied in Model selection and hyper-parameter tuning. Finally, the OSA severity class was assigned to each participant based on the AHI_pred_ and the canonical clinical thresholds.

### Performance analysis

Independently by the sleep/wake score used, our method was evaluated with regards to the AHI estimation, the screening capability and the OSA severity estimation. The AHI estimation was evaluated via Spearman correlation coefficient and Bland-Altman plot between the reference AHI and the AHI_pred_. The Spearman correlation coefficient was used because the reference AHI and AHI_pred_ were heteroscedastically related, i.e. the variability of AHI_pred_ was unequal across the range of values of AHI (p < 0.01 for the Breusch-Pagan test^60^). In addition, the average AHI estimation error for the different OSA severities was also reported to complement the correlation analysis. Participants with a difference between the reference AHI and the AHI_pred_ >30 events/h or <−30 events/h were defined as, respectively, considerably underestimated and considerably overestimated. The 30 events/h threshold was determined based on the limits of agreement of our AHI estimation in addition to 30 events/h being the minimum difference to misclassify a person without any respiratory event as a severe OSA (and vice versa). The screening capability of the AHI_pred_ was quantified using standard statistical measures of the performance for binary classification, such as specificity and sensitivity, and receiver operating characteristic curves for each OSA severity. Confusion matrices were employed to evaluate the OSA severity estimation performance.

## Data Availability

The UCD and the Apnea-ECG datasets are well known datasets in the OSA research field and available respectively at https://physionet.org/physiobank/database/ucddb/ and at https://physionet.org/physiobank/database/apnea-ecg/. The SOMNIA and the HealthBed datasets are built to facilitate future research in sleep medicine. The data are available from the Sleep Medicine Centre Kempenhaeghe upon request, by providing a research protocol to be approved by the institutional research board. Specific restrictions apply to the availability of the data collected with sensors not comprised in the standard PSG set-up, since these sensors are used under license and are not publicly available. These data are however available from the authors upon reasonable request and with permission of the licensors. The Auto-PSG dataset was created through an unrestricted grant by Philips Electronics North America to test an automatic sleep scoring software. The dataset can be used for research purposes within Philips Research. Philips Electronics holds the ownership of the dataset and it can be shared only if a loan and license agreement with Philips Electronics North America is in place.
